# Cardioprotective Effects of Palmitoleic Acid (C16:1n7) in a Mouse Model of Catecholamine-Induced Cardiac Damage Are Mediated by PPAR Activation

**DOI:** 10.3390/ijms222312695

**Published:** 2021-11-24

**Authors:** Iris Rosa Betz, Sarah Julia Qaiyumi, Madeleine Goeritzer, Arne Thiele, Sarah Brix, Niklas Beyhoff, Jana Grune, Robert Klopfleisch, Franziska Greulich, Nina Henriette Uhlenhaut, Ulrich Kintscher, Anna Foryst-Ludwig

**Affiliations:** 1 Center for Cardiovascular Research, Institute of Pharmacology, Charité-Universitätsmedizin Berlin, Corporate Member of Freie Universität Berlin and Humboldt-Universität zu Berlin, 10115 Berlin, Germany; iris.betz@charite.de (I.R.B.); sarah.qaiyumi@charite.de (S.J.Q.); madeleine.goeritzer@charite.de (M.G.); arne.thiele@charite.de (A.T.); sbrix@gmx.de (S.B.); niklas.beyhoff@charite.de (N.B.); jana.grune@charite.de (J.G.); ulrich.kintscher@charite.de (U.K.); 2 Berlin Institute of Health, Emergency Department Campus Benjamin Franklin, Charité-Universitätsmedizin Berlin, Corporate Member of Freie Universität Berlin and Humboldt-Universität zu Berlin, 12203 Berlin, Germany; 3 DZHK (German Centre for Cardiovascular Research), Partner Site Berlin, 10785 Berlin, Germany; 4 Department of Veterinary Pathology, College of Veterinary Medicine, Freie Universität Berlin, 14163 Berlin, Germany; Robert.Klopfleisch@fu-berlin.de; 5 German Center for Environmental Health GmbH, Institute for Diabetes and Cancer (IDC), 85764 Munich, Germany; franziska.greulich@tum.de (F.G.); henriette.uhlenhaut@helmholtz-muenchen.de (N.H.U.); 6 Metabolic Programming, School of Life Sciences Weihenstephan, Technische Universitaet Muenchen (TUM), 85354 Freising, Germany; 7 DZHK (German Centre for Cardiovascular Research), Partner Site Munich, 13125 Berlin, Germany

**Keywords:** palmitoleic acid (C16:1n7), lipokine, catecholamine, cardiac damage, PPAR, cardioprotective effects

## Abstract

Palmitoleic acid (C16:1n7) has been identified as a regulator of physiological cardiac hypertrophy. In the present study, we aimed to investigate the molecular pathways involved in C16:1n7 responses in primary murine cardiomyocytes (PCM) and a mouse model of isoproterenol (ISO)-induced cardiac damage. PCMs were stimulated with C16:1n7 or a vehicle. Afterwards, RNA sequencing was performed using an Illumina HiSeq sequencer. Confirmatory analysis was performed in PCMs and HL-1 cardiomyocytes. For an in vivo study, 129 sv mice were orally treated with a vehicle or C16:1n7 for 22 days. After 5 days of pre-treatment, the mice were injected with ISO (25 mg/kg/d s. c.) for 4 consecutive days. Cardiac phenotyping was performed using echocardiography. In total, 129 genes were differentially expressed in PCMs stimulated with C16:1n7, including Angiopoietin-like factor 4 (*Angptl4*) and Pyruvate Dehydrogenase Kinase 4 (*Pdk4*). Both Angptl4 and Pdk4 are proxisome proliferator-activated receptor α/δ (PPARα/δ) target genes. Our in vivo results indicated cardioprotective and anti-fibrotic effects of C16:1n7 application in mice. This was associated with the C16:1n7-dependent regulation of the cardiac PPAR-specific signaling pathways. In conclusion, our experiments demonstrated that C16:1n7 might have protective effects on cardiac fibrosis and inflammation. Our study may help to develop future lipid-based therapies for catecholamine-induced cardiac damage.

## 1. Introduction

Heart failure (HF) remains a leading cause of cardiovascular mortality and although new therapeutic approaches were recently established to treat HF with reduced ejection fraction (HFrEF), and to some extent, HF with preserved ejection fraction (HFpEF) [1], the demand for new pharmacological strategies to treat those diseases remains still unmet [2]. The development of HF has been linked to the exuberated activation of the sympathetic nervous system, leading to the persistent release of catecholamines [3]. Accelerated and continuous catecholamine load on cardiomyocytes causes increased cardiac contractility, as well as hypoxia, inflammation and cardiac apoptosis [4]. Thus, in our previous study, we used a model of catecholamine-induced cardiac damage to investigate the development of cardiac fibrosis, inflammation and apoptosis after isoproterenol (ISO) application to 129 sv mice [5,6,7,8].

Apart from their cardio-excitatory effects, mediated mostly by adrenergic β-1 receptors on cardiomyocytes, catecholamines induce activation of lipolysis in white adipose tissue (WAT), contributing to the increase in circulating lipids and non-esterified fatty acids (NEFAs) [9]. NEFAs are considered as efficient fuel for cardiac metabolism, and enhanced lipid uptake and FA oxidation were linked with the development of physiological cardiac hypertrophy, induced by exercise or pregnancy [10,11]. In the case of disturbed FA metabolism and misbalance between NEFAs uptake and utilization (for instance, in the case of mitochondrial dysfunction and metabolic switch of the cardiomyocytes to pronounced glucose metabolism), lipotoxicity can occur [12,13]. Importantly, some saturated NEFAs and their metabolites, such as palmitic acid (C16:0) or myristic acid (C14:0), are known to induce proinflammatory and pro-fibrotic effects when applied to mice or to cardiomyocytes in cell culture experiments [14,15,16]. Distinct monosaturated NEFAs (MUFAs), such as oleic acid (C18:1) [16], or polyunsaturated fatty acids (PUFAs) such as eicosapentaenoic acid (EPA) and docosahexaenoic acid (DHA), display cardioprotective effects, reviewed by [17]. In contrast, the cardio-modulatory role of palmitoleic acid (C16:1n7) are discussed controversially. Previously, C16:1n7 was identified as a cardioprotective MUFA involved in the development of physiological hypertrophy in mice and snakes [18,19]. The putative cardioprotective effect linked with C16:1n7 in humans remains controversial. Some clinical trials indicated no significant correlation between plasma, serum, WAT or erythrocyte membrane levels of C16:1n7 and increased cardiovascular mortality in patients suffering from ischemic heart disease or HF [20,21,22], as reviewed by [23], whereas other studies identified a positive correlation [24,25], as reviewed by [23]. Other clinical trials implicated a cardioprotective effect of palmitoleic acid [26]. In addition, C16:1n7plasma levels have been linked to the development of metabolic syndrome and obesity [27]. On the other hand, a study performed by Souza et al. indicated an inverse correlation between the development of obesity and C16:1n7 serum level [20]. C16:1n7 was also demonstrated to correlate with BMI and HDL cholesterol levels, but not with serum LDL cholesterol [28]. Results published by Stefan et al. indicate a positive correlation between circulating cis-C16:1n7 and increased insulin sensitivity [29]. Importantly, C16:1n7 was also recognized as an adipokine with strong protective metabolic effects, such as increased insulin sensitivity and decreased hepatic lipid accumulation [30,31]. 

In the present study, we aimed to identify molecular pathways involved in the putative cardioprotective effects of C16:1n7 application in vitro, using isolated adult murine cardiomyocytes, and in vivo, with the model of ISO-induced cardiac damage. Our data indicate that C16:1n7 facilitates some anti-fibrotic and anti-inflammatory effects in vivo, mediated by the specific activation of key regulators of cardiac metabolism: peroxisome proliferator-activated receptors (PPAR) α/δ.

## 2. Results

### 2.1. C16:1n7 Induces PPARα/δ-Specific Gene Expression Profile in the Primary Adult Murine Cardiomyocytes (PCMs)

Our previous studies identified C16:1n7 as a new adipokine involved in the regulation of physiological cardiac hypertrophy in vivo [19], but the molecular mechanism involved in this hypertrophic process is still unclear.

**Figure 1:**
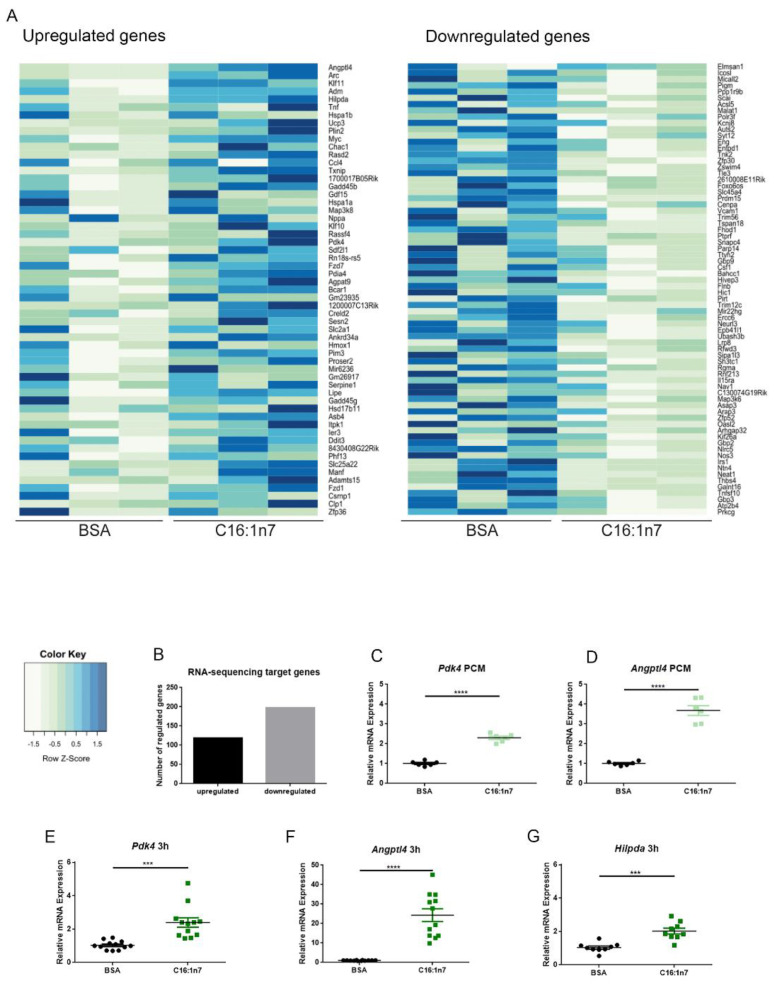
Palmitoleic acid (C16:1n7)-derived gene expression regulation of primary adult cardiomyocytes (PCMs) and HL-1 cardiomyocytes. (**A**) RNA-Seq results of PCMs stimulated with 470 μM C16:1n7 or 10% FFA-free BSA control for 3 h. Heatmap of 50 significantly up- and downregulated genes; mean base expression, 50; fold change filter, 1.5×; FDR-adjusted *p*-value of 0.1; >100 base expressions per sample; *n* = 3. (**B**) Cumulative results of all C16:1n7-regulated genes, detected using RNA-Seq analysis; mean base expression, 50; fold change filter, 1.5×; FDR-adjusted *p*-value of 0.1; *n* = 3. (**C**, **D**) Confirmatory experiments performed on PCMs treated with C16:1n7 470 μM C16:1n7 or 10% FFA-free BSA control for 3 h in experiments perform parallel to RNA-Seq analysis. Relative expressions of *Angptl4* and *Pdk4* are shown, as indicated. (**E**–**G**) Confirmatory experiments performed on HL-1 cardiomyocytes treated with C16:1n7 470 μM C16:1n7 or 10% FFA-free BSA control for 3 h. Relative expressions of *Angptl4*, *Pdk4* and *Hilpda* are shown, as indicated. *Angptl4*: Angiopoietin-like factor 4; *Pdk4*: Pyruvate Dehydrogenase Kinase 4, *Hilpda*: Hypoxia Inducible Lipid Droplet Associated. Data are presented as mean ± SEM. *n* = 3, *n* = 3; *** *p* < 0.001, **** *p* < 0.0001 as analyzed by two-way ANOVA followed by Bonferroni’s post-hoc test.

In the present study, we aimed to identify a specific set of genes, regulated in PCMs in a C16:1n7-dependent manner. PCMs were isolated from mice using a Langendorff system and collagenase type 2 digestion procedure, as described in the Methods section. After Ca^2+^ build-up, the PCMs were stimulated with 470 μM C16:1n7 or a vehicle for 3 h. The amount of palmitoleic acid used in all in vitro experiments was chosen as an equivalent concentration to the serum levels of that MUFA, measured in mice in our previous studies [19,32]. Afterwards, the PCMs were harvested, and total RNA was isolated. RNA-Seq was performed using an Illumina HiSeq sequencer, as described in the Material and Methods section. In total, 129 genes were differentially expressed in the PCMs stimulated with C16:1n7 compared to control cells, including the highly regulated Angiopoietin-like factor 4 (*Angptl4*) and Pyruvate Dehydrogenase Kinase 4 (*Pdk4*) (Figure 1A,B). In addition, AKT (Protein kinase B (PKB) signaling pathway downstream targets, e.g., Breast Cancer Anti-estrogen Resistance 1 (*Bcar1*) and Provirus integrating site Moloney murine leukemia virus 3 (*Pim3*), were also differentially regulated (Figure 1A). Importantly, AKT kinase was recognized as a main regulator of physiological cardiac hypertrophy in our previous study [19,33]. Both *Angptl4*, known for its anti-inflammatory and anti-fibrotic properties, and *Pdk4*, involved in mitochondrial lipid utilization, are well-known PPARα/δ target genes and therefore of special interest for further analysis (Figure 1A). Importantly, PPARs belong to the family of nuclear hormone receptors, acting as transcriptional factors, and are considered as key regulators of FA and glucose metabolism. Confirmatory experiments of RNA-Seq data were performed in PCMs and HL-1 cardiomyocytes. Both PCMs and HL-1 cells were stimulated with 470 μM C16:1n7 or a vehicle for 3 h, as described previously. qRT-PCR analysis indicated significant regulation of *Pdk4* and *Angptl4* (Figure 1C–F). In addition, HL-1 cells stimulated with C16:1n7 showed increased expression of *Hilpda* (hypoxia inducible lipid droplet associated protein), also a PPARα target gene, and belonging to the 25 most highly upregulated genes in our RNA-Seq experiment. In addition, we performed lactate dehydrogenase (LDH) assay to prove PCMs viability. The LDH assay revealed an increased survival of the C16:1n7-treated PCMs when compared to vehicle-treated control cells (data not shown), which indicated no obvious lipotoxic effects of palmitoleic acid in our experiments.

**Figure 2:**
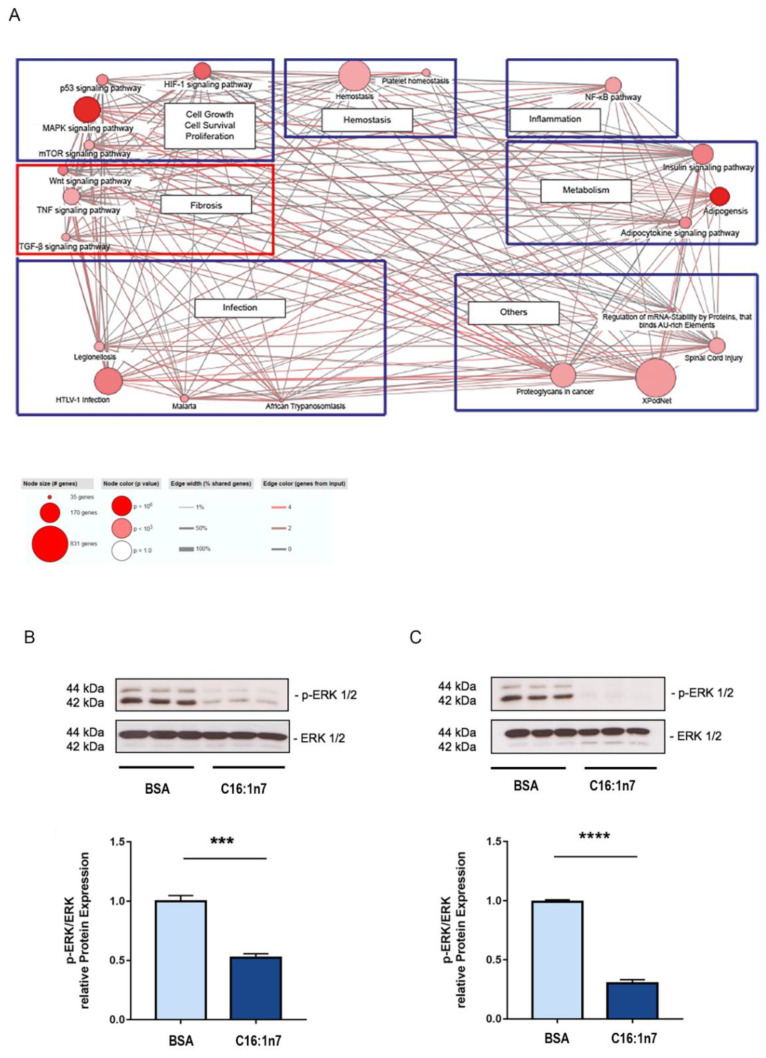
(**A**) CPDB pathway analysis of RNA-Seq data presented in Figure 1. For details, please see Materials and Methods. (**B**,**C**) Western blot analysis of ERK1/2 phosphorylation in HL-1 cardiomyocytes, stimulated with 471 µM C16:1n7 for 5 and 15 min. Data are presented as mean ± SEM. *n* = 3, *n* = 3; *** *p* < 0.001, **** *p* < 0.0001, as analyzed using unpaired *t*-tests.

Next, further RNA-Seq gene set analysis was performed using the CPDB website (http://cpdb.molgen.mpg.de/MCPDB, accessed on 30 March 2017). Importantly, CPDB analysis identified key metabolic pathways belonging to glucose and lipid metabolism, as well as fibrosis-related pathways (including the WNT (Wingless and Int-1) and TGFβ (Transforming growth factors) pathways) that were influenced by C16:1n7 treatment (Figure 2). In addition, proliferation, hemostasis and NF-κB pathways (inflammation) were also significantly regulated. Taken together, cardiomyocytes stimulated ex vivo with C16:1n7 were characterized by selective upregulation of PPARα/δ target genes, such as *Angptl4* and *Pdk4*, both involved in the regulation of FA uptake and oxidation. In addition, those experiments indicated the putative anti-inflammatory and anti-fibrotic action of C16:1n7, as both NF-κB and fibrosis-associated signaling pathways (WNT and TGFβ) seemed to be regulated after C16:1n7 stimulation in cardiomyocytes. Another pathway that was significantly regulated in C16:1n7-treated PCMs was the mitogen-activated protein kinase (MAPK) pathway. As PPARα activity was reported to be regulated in cardiomyocytes by extracellular signal regulated protein kinase 1 and 2 (ERK1/2), belonging to the MAPK signaling pathway, we investigated the activity/phosphorylation of ERK1/2 in those cells [34,35]. In accordance, C16:1n7 stimulation led to a significant inhibition of ERK1/2 phosphorylation in HL-1 cardiomyocytes under C16:1n7 stimulation (Figure 2B,C). To analyze the putative hypertrophic effects of C16:1n7 stimulation on cardiomyocytes, downstream of AKT/MAPK pathways, we next focused on the Forkhead Box Protein O (FOXO). The FOXO family of transcription factors is involved in the regulation of glucose and lipid metabolism, as well as cell cycle progression in cardiomyocytes upon IGF1 stimulation [36]. Phosphorylation of FOXO leads to its proteasomal degradation under the activation of the IGF1/insulin/AKT pathway. As demonstrated in Supplementary Figure S1A (see Supplementary Materials), C16:1n7 stimulation led to pronounced phosphorylation/inhibition of FOXO1 and FOXO3 in cardiomyocytes. That could explain, at least in part, the putative pro-hypertrophic effects of the MUFA observed previously [19].

### 2.2. C16:1n7 Displays Anti-Fibrotic and Anti-Inflammatory Action in the Model of ISO-Induced Cardiac Damage in Mice

**Figure 3:**
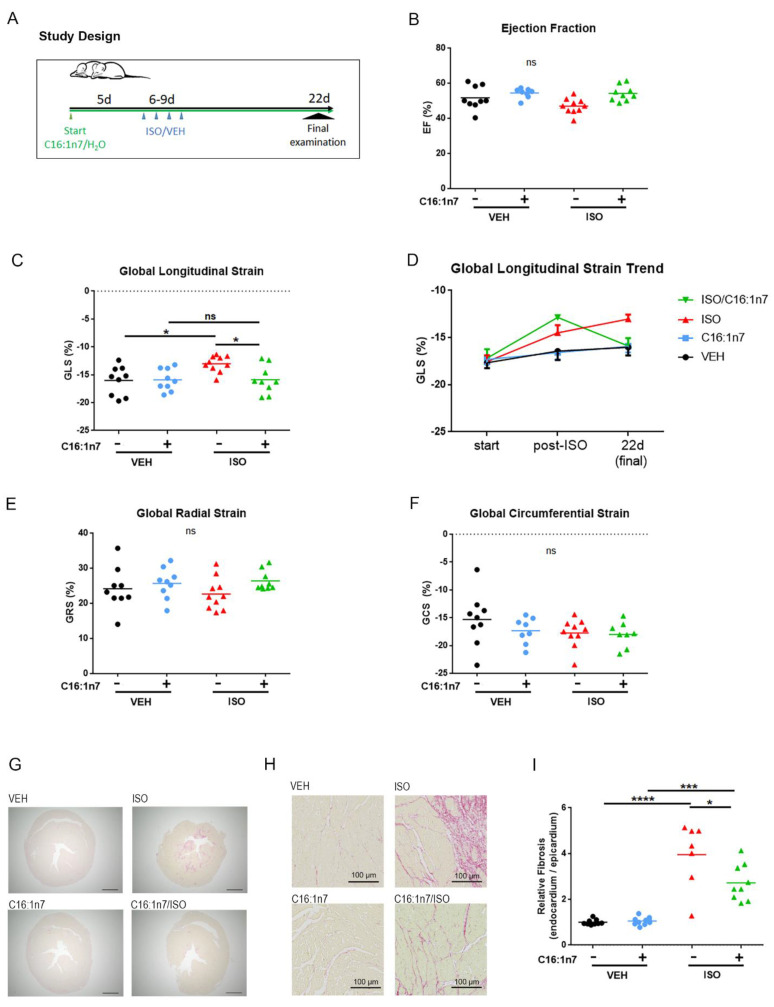
C16:1n7 application to mice protects against ISO-induced cardiac damage. (**A**) Experimental design. The 129 sv wt mice were daily orally supplemented with C16:1n7 or vehicle as the control. After 5 days (pretreatment), the mice received additional s. c. ISO/Veh application for 4 consecutive days. At baseline, the day after the last ISO application and during the final examination (day 22), the animals underwent echocardiographic analysis. (**B**–**F**) Results from the echocardiographic analysis of the mice: (**B**) left ventricular ejection fraction (EF); (**C**) global longitudinal peak strain (GLS); (**D**) GLS trend; (**E**) global radial peak strain (GRS); (**F**) global circumferential peak strain (GCS). (**G**–**I**) Analysis of cardiac fibrosis using Picrosirius red staining. (**G**) Representative images are shown. Scale bar = 1 mm. (**H**) Representative images of subendocardial fibrosis are shown. Scale bar = 100 μm, as indicated. (**I**) Relative content of subendocardial collagen; data presented as a ration between endocardial and epicardial fibrosis, as indicated. Data are presented as mean ± SEM. *n* = 8–10 per group, or as indicated. ns: p>0.05, * *p* < 0.05, *** *p* < 0.001, **** *p* < 0.0001 as analyzed by two-way ANOVA followed by Tukey’s post-hoc test.

To investigate the putative cardioprotective effects of C16:1n7 application in vivo, we used an established model of ISO-induced cardiac damage, described previously [5,6,7,8]. Briefly, 129 sv wt mice were daily orally supplemented with C16:1n7 or the vehicle [19,31]. After 5 days (pre-treatment), mice received additionally subcutaneous ISO/Veh application for four consecutive days (Figure 3A). At baseline, the day after the last ISO application and during the final examination (day 22), the animals underwent echocardiographic analysis, as described in the Materials and Methods section. The results from echocardiographic analysis are depicted in Figure 3B–F and in Table 1. ISO treatment led to the significant impairment of the global longitudinal peak strain (GLS, Figure 3C,D), an effect significantly improved in mice supplemented with C16:1n7 (Figure 3C,D), but not C18:1n9 (Supplementary Figure S1B). Interestingly, trend analysis of the GLS throughout the study (Figure 3D) indicated significant improvement of the GLS parameter only after 22 days of C161n7 application. Directly after ISO treatment, no significant differences between ISO and C16:1n7/ISO groups were detected. This implies a long-term C16:1n7-directed remodeling processes in the myocardium, resulting in complete GLS normalization at the end of the study (Figure 3C,D), which cannot be seen with a supplementation of C18:1n9 (Supplementary Figure S1B). In accordance with our previous results, ISO application did not affect ejection fraction or fractional shortening (Figure 3B and Table 1), and had no effect on the other strain parameters (global radial peak strain and global circumferential peak strain) (Figure 3E,F) [5,8]. In addition, other echocardiographic parameters, such as diastolic left ventricular (LV) posterior wall thickness (LVPWd), diastolic LV internal diameter (LVIDd) and diastolic septum thickness (IVSd), as well as LV mass and heart weight, were not significantly different between both ISO-treated groups (Table 1).

As changes in GLS in this mouse model were shown to be strongly associated with cardiac fibrosis [5,6,8], we next investigated the fibrotic changes studied in myocardial paraffin sections of the mice. As expected, ISO treatment led to the development of cardiac fibrosis (Figure 3G–I), and that process was significantly reduced by the C16:1n7 treatment, suggesting a putative anti-fibrotic action of C16:1n7. 

**Figure 4:**
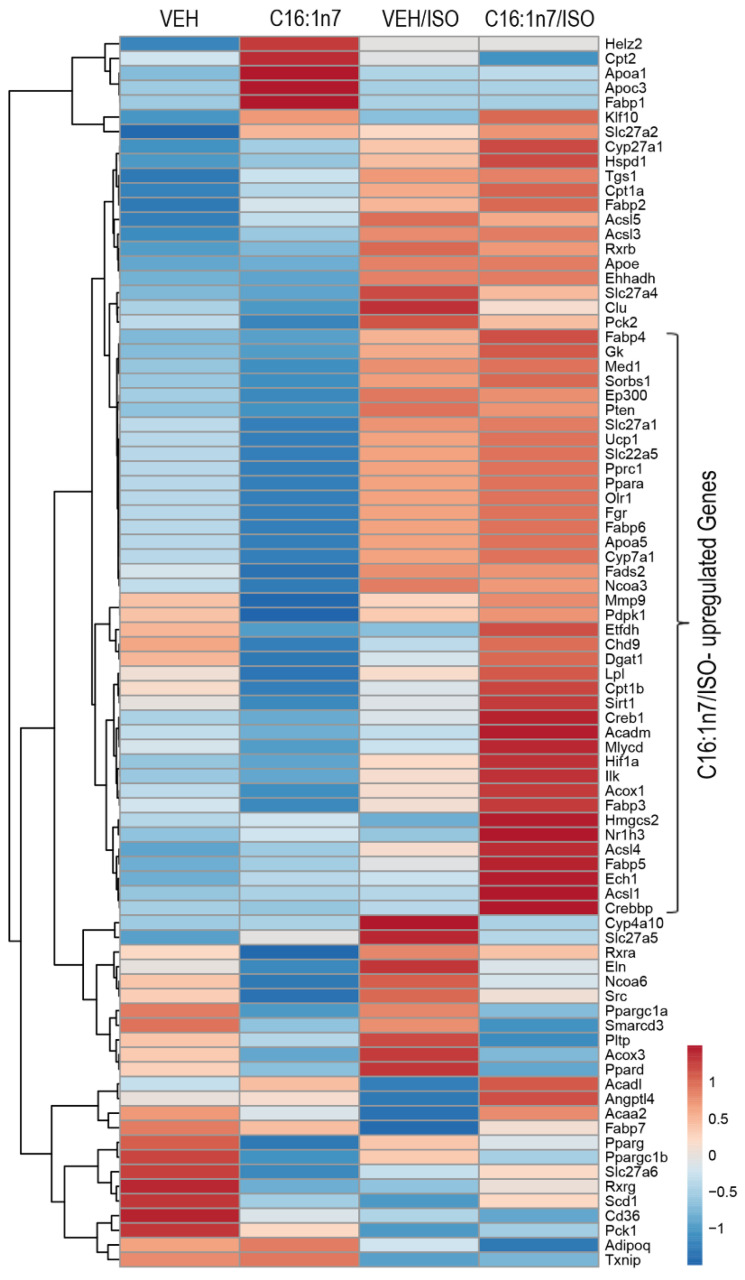
C16:1n7 mediates its cardioprotective effects activating PPAR target gene expression. qRT-PCR-based cardiac array of PPAR target genes, *n* = 3 per group. A hierarchical clustering and heatmap of expression values with color-coded row z-scores is shown.

As the main regulatory effect of C16:1n7 in PCMs was connected to activation of PPAR-target genes, such as *Angptl4* or *Pdk4*, we next performed a PPAR-directed expression array of cardiac tissue samples from the mice treated with C16:1n7 or the vehicle control (Figure 4). As depicted in Figure 4, the analysis of the cardiac gene expression indicated a set of around 45 PPAR-target genes, which were strongly upregulated in the C16:1n7/ISO-treated group when compared to ISO-treated mice (Figure 4). The C16:1/ISO-induced genes are PPARαδ target genes involved in FA transport und uptake, such as several FA transporters and FA-binding proteins (*Fabp 3, 5* and *6*); *Lpl* (lipoprotein lipase), multiple genes regulating mitochondrial FA oxidation, such as *Acox1* (Acyl-CoA Oxidase 1), *Cpt1β* (carnitine palmitoyltransferase 1 β), *Acls1* and *4* (acyl-CoA Synthetase Long Chain Family Member 1 and 4), *Acadm* (Acyl-CoA Dehydrogenase Medium Chain), *Mlycd* (malonyl-CoA decarboxylase) and *Hmgcs2* (HMG-CoA synthase), or involved in the regulation of mitochondrial function, such as *UCP1* (Uncoupling Protein 1) and *Etfdh* (Electron Transfer Flavoprotein Dehydrogenase). Interestingly, a small cluster of genes was exclusively activated by C16:1n7, including *Fabp1*, *Cpt1* as well as *Apoa1* and *Apoc3* (Apolipoprotein C3 and Apolipoprotein A-I), known components of triglyceride (TG)-rich lipoproteins (TRLs) and HDL in plasma [37,38]. We also identified a small cluster of ISO-induced genes, such as *Pparδ* and *Slc27a4* and *5* (Solute Carrier Family 27 Member 4 and 5), belonging to the FA transporters across the plasma membrane, as well as *Clu* (Clusterin), a cytosolic chaperone, induced by stress conditions, and linked with cell death/tumor progression [39]. Taken together, orally administered C16:1n7 was able to reduce pro-fibrotic and pro-inflammatory effects in the model of isoproterenol-induced cardiac damage. Those effects are mediated, at least in part, by the cardiac activation of key regulators of cardiac glucose and FA metabolism, PPARα and PPARδ.

## 3. Discussion

Our study demonstrated for the first time that C16:1n7 leads to the activation of several PPARα/δ-specific genes in the PCMs. Anti-inflammatory and cardioprotective effects of PPARα/δ are well documented (reviewed by Francis et al. [40]). Cardioprotective actions of PPARα, besides the lipid-lowering hepatic effects, are related to enhanced expression and activity of enzymes and transporters, mediating lipid uptake and mitochondrial FA oxidation [40]. Importantly, due to the elevated energy demand during the development of HF, the persistent ability of the heart to utilize FA seems to be of major importance. In accordance, the results from our study indicate a strong expressional regulation of the genes linked with mitochondrial FA transport and FA oxidation in PCMs, HL-1 cardiomyocytes, as well as in vivo in the cardiac tissue of C16:1n7/ISO-treated mice.

Contrary to PPARα, the cardioprotective role of PPARδ, a second highly expressed isotype of PPAR in the myocardium, is linked with the cardiac modulation of inflammation and fibrosis. As reviewed by Magadum and Engel [41], PPARδ was shown to inhibit indirectly cardiac apoptosis and inflammation, to reduce fibrosis and to accelerate angiogenesis, interfering with cardiac NF-κB/PDK1 (3-phosphoinositide-dependent protein kinase)/Akt and GSK3 (Glykogen Synthase Kinase 3), as well as Wnt pathways [41]. Activation of PPARδ using synthetic ligands attenuated C16:0-induced apoptosis in neonatal cardiomyocytes by inhibiting an increase in interleukin 6 levels [42]. Importantly, as discussed previously, MUFAs, PUFASs and other lipid metabolites (e.g., prostacyclin and 15-hydroxyeicosatetraenoic acid (15-HETE)) were demonstrated to activate PPARs in vitro and in vivo [40,41]. In a previous study performed by Brown et al., C16:1n7 was shown to induce hepatic PPARδ activity [43]. Not only palmitoleic acid, but also other MUFAs and PUFAs were demonstrated to induce PPARs activity in vitro, predominantly stimulating PPARα [44]. On the other hand, transcriptional activity of PPARα and PPARδ seems to be, at least in part, redundant, as demonstrated in the study published by Muoio et al. [45], using PPARα-deficient mice. In addition, our previously published experiments using C16:1n7-stimulated HL-1 cardiomyocytes followed by nuclear fractionation implicated nuclear localization of C16:1n7 6 h after palmitoleic acid was added to the cell culture medium [19]. This experiment suggested rather a direct activation of PPARs with C16:1n7 in the nucleus of cardiomyocytes.

In addition to PPAR signaling pathways, our RNA-Seq experiments pointed towards the regulation of TGF-β/WNT-dependent genes, as well as the regulation of NF-κB-related genes. WNT signaling was shown to be activated by C16:1n7 stimulation in vitro [46]. On the other hand, palmitoleic acid-derived WNT activation was demonstrated to regulate AKT kinase activity. We were recently able to demonstrate that AKT activation induces cardiac remodeling and development of physiological cardiac hypertrophy in vivo and in vitro [19,33]. In addition, it is well established that proinflammatory transcription factor NF-κB can be selectively inhibited via trans-repression by activated PPARs, which explain, at least in part, the well-documented anti-inflammatory action of PPAR agonists [47,48]. Our in vivo study indicated that oral administration of C16:1n7 improved cardiac function in the model of ISO-induced cardiac damage. Our previous experiments indicated that ISO application to mice led to the enhanced apoptosis and inflammation in the heart of the mice as soon as a few days after the last ISO injection [5,8]. The apoptotic and proinflammatory effects were then followed by augmented pro-fibrotic cardiac response [5,8]. We focused on the putative long-term cardioprotective effects of C16:1n7 supplementation and were able to demonstrate that palmitoleic acid improved cardiac function (GLS), as well as cardiac fibrosis at the end of the study. Interestingly, short-term application did not improve cardiac function, measured by echocardiography (GLS-rate), indicating that the preventive properties of C16:1n7 treatment in the heart were not mediated by direct effects of palmitoleic acid application on apoptosis or hypoxia in myocardium under increased catecholamine load. More surprising is the complete recovery of cardiac function, observed after the study was terminated. One possible explanation would be a regulation of several regenerative signaling pathways, described in the context of PPARδ activation. In accordance with this hypothesis, PPARδ was shown to mediate survival, proliferation, differentiation and angiogenesis, as well as mammalian regeneration of the skin, bone and liver [41]. As discussed above, protective effects of C16:1n7 treatment may be mediated by the PPARδ activation of TGFβ/WNT signaling, as well as the inhibition of NF-κB signaling pathways.

PPARα activity seems to be regulated in an ERK1/2-specific manner, and our experiments indicated that HL-1 cardiomyocytes stimulated with C16:1n7 are characterized by a significant reduction in ERK1/2 phosphorylation status. This is in accordance with a previously published animal study using a transverse aortic constriction (TAC) model in mice and according to in vitro experiments performed in rat neonatal cardiomyocytes under phenylephrine (PE) stimulation (pathological cardiac hypertrophy) or insulin-like growth factor 1 (IGF-1) treatment (physiological cardiac hypertrophy) [34,35]. During the development of HF in the TAC model in mice and in vitro, in rat neonatal cardiomyocytes under PE stimulation, PPARα expression and PPARα transcriptional activity were strongly inhibited, which was associated with a significant phosphorylation and activation of ERK1/2 [34,35]. On the other hand, when stimulated with IGF1, rat neonatal cardiomyocytes showed increased PPARα activity, linked with a significant de-phosphorylation of both ERK1/2. The findings confirmed our hypothesis that C16:1n7 is involved in the regulation of physiological cardiac adaptation. Importantly, significantly increased ERK1/2 activation/phosphorylation was recently linked to the development of cardiac fibrosis in the model of ISO-induced cardiac damage in mice [49]. However, we observed rather short-term effects of C16:1n7 stimulation on ERK1/2 phosphorylation, and could not exclude PPAR-independent effects of FA on ERK1/2 activity.

In the past, pharmacological activation of PPAR (especially PPARγ) was reported to negatively modify cardiovascular risk and morbidity in heart failure patients. As shown by Lago et al. [50], heart failure patients treated with Pioglitazone (a PPARγ-agonist) had an increased risk of acute cardiac decompensation, but not overall cardiovascular mortality. This might be explained by an increased fluid retention induced by renal glitazone actions [50]. No such side effects are known for PPARα and delta ligands.

C18:1n9 was not able to show the same benefit as C16:1n7 in our ISO model of cardiac fibrosis. This might be due to a specific activation pattern of PPARs only mediated by C16:1n7. Not all fatty acids bind to PPARs with the same affinity [51]. Further research is necessary to elucidate this differential effect of MUFAs, representing a limitation of the present study.

Another limiting factor is that we analyzed most of our findings in cardiomyocytes. Cardiomyocytes are not the main cells responsible for the development of cardiac fibrosis. Nevertheless, stressed cardiomyocytes are able to activate proinflammatory and pro-fibrotic pathways [52]. Our findings in the ISO model verified the validity of our sequencing findings and proved our hypothesis that C16:1n7 is able to attenuate cardiac fibrosis. However, further experiments including fibroblasts and immune cells could help to shed more light onto the mechanisms behind the anti-fibrotic properties of C16:1n7.

Further experiments using C16:1n7 application in the ISO model applied to cardiomyocyte-specific PPARα-KO and PPARδ-KO mice could allow a better understanding of the molecular mechanism mediated by palmitoleic acid supplementation in the future. In addition, other cardioprotective signaling pathways, beyond PPAR activation, could also contribute to those effects.

In summary, we demonstrated putative cardioprotective effects of C16:1n7 supplementation in vivo, likely mediated by the cardiac activation of PPARα and PPARδ. In the present study, we could not exclude that some additional beneficial C16:1n7 actions are mediated by non-cardiac processes. Nevertheless, based on the study performed by Cao et al. [30], C16:1n7 was accepted by the FDA for the preventive treatment of several metabolic diseases, such as obesity-induced insulin resistance and T2DM. Our data indicate that palmitoleic acid could also be important for the preventive treatment of cardiometabolic disorders, such as cardiac fibrosis and inflammation.

## 4. Materials and Methods

### 4.1. Isolation of Adult Murine Primary Cardiomyocytes (PCMs)

Adult murine primary cardiomyocytes were isolated from C57Bl/6J wt mice, as described before [53]. Mice were injected with heparin (500 IE) and afterwards anesthetized using isoflurane and sacrificed by cervical dislocation. Hearts were removed, cannulated and mounted onto a Langendorff perfusion apparatus. Next, each heart was perfused retrogradely with a Ca^2^⁺-free perfusion buffer and subsequently with a buffer containing 1250 U/l Collagenase Type 2 (Worthington, NJ, USA) and 0.01 mM Ca^2^⁺, until the heart was pale and soft. Next, the heart was cut off the cannula, minced and filtered through a 140 µm nylon net filter (Merck Millipore, Germany). A Ca^2^⁺-free perfusion buffer containing 10% Fetal Bovine Serum (FBS) was added to stop the collagenase activity, and the sample was centrifuged for 1 min at 500 rpm at 25 °C [54]. Afterwards, PCMs were gravity-sedimented and resuspended in perfusion buffer containing gradually increasing Ca^2^⁺ concentrations (0.1 mM Ca^2^⁺; 0.5 mM Ca^2^⁺; 1 mM Ca^2^⁺) without 2,3 butanedione monoxime for 10 min [54]. Finally, isolated PCMs were centrifuged, and the pellets were either re-suspended with Claycomb medium for further assays or frozen at −80°C for RNA analysis. The purity and viability of PCMs was analyzed using light microscopy and immunofluorescence-based staining for desmin/CD31/α-Actinin and vimentin (data not shown).

### 4.2. Animal Experiments

The ISO-induced model of cardiac damage was described previously [5,6,7,8]. Briefly, 7–9-week-old 129 sv male wt mice (Janvier Labs, Le Genest-Saint-Isle, France) were used. Five days prior to the first ISO injection and throughout the study, the mice were orally supplemented with C16:1n7 and C18:1n9 (300 mg/kg/day) or water (Vehicle), as described previously [19,31]. After the first 5 days, mice were subcutaneously injected with ISO (25 mg/kg body weight, dissolved in saline) or saline for 4 consecutive days (Figure 3A). At baseline, directly after the last ISO application and during final examination, the mice underwent echocardiographic examination, as described before [5,6,7,8]. Briefly, mice were examined on a Vevo 3100 Imaging System equipped with a 30 MHz linear transducer (MX400; FUJIFILM VisualSonics Inc., Toronto, ON, Canada). Inhalation anesthesia with 1–3% isoflurane was used. B-Mode and M-Mode images were obtained and analyzed using Vevo LAB analysis software (FUJIFILM VisualSonics Inc., Canada). Global myocardial peak strain (rate) was semi-automatically assessed in B-mode images acquired using Vevo Strain Software with integrated two-dimensional speckle-tracking algorithm. After 22 days mice, were anesthetized by isoflurane and sacrificed by cervical dislocation. Sample sizes are indicated in each figure legend.

### 4.3. Cell Culture Experiments with HL-1 Cardiomyocytes and PCMs

Mouse HL-1 cardiomyocytes, kindly provided by W.C. Claycomb (Louisiana State University, Baton Rouge, LA, USA) were cultivated, as described previously [19]. After a 24 h starvation period (0.5% FBS), cells were stimulated with 10% FFA-free bovine serum albumin (BSA) or 470 μM C16:1n7, dissolved in 10% FFA-free BSA for 3 h (mRNA expression), as described before [19]. C16:1n7 was used in equimolar serum concentrations, as estimated by FA profiling (470 μM) performed previously [19]. Analog experiments were performed with PCMs direct after isolation procedure.

### 4.4. PCMs-RNA Sequencing and Pathway Analysis

The RNA sequencing was performed in cooperation with Franziska Greulich and Nina Henriette Uhlenhaut. Briefly, total RNA was extracted using RNeasy Micro Kit according to the manufacture’s protocol (Qiagen, Hilden, Germany). An RNA sequencing library was created using the Illumina TruSeq RNA Library Prep Kit according to the manufacturer’s protocol (https://emea.illumina.com/products/by-type/sequencing-kits/library-prep-kits/truseq-rna-v2.html?langsel=/de, Pub. No. 770-2009-039 Current as of 17 November 2014). Quality control was performed using Illumina HiSeq^®^. The library was sequenced using Illumina HiSeq^®^ 1000. The analysis was performed using R (www.R-project.org). A gene was considered differentially expressed if it reached a 1.5-fold change, passed a false discovery rate (FDR) of 0.1 and had mean base expression of at least 100. The pairwise comparison of C16:1n7 versus BSA-treated PCMs revealed 129 differently expressed genes. The 50 highly regulated up- and downregulated genes are depicted in a heatmap. Original RNAseq data are available in the supplementary file (Table S1).

### 4.5. Pathway Analysis Using ConsensusPath DB (CPDB)

Sequencing analysis was extended using over-representation analysis by Herwig et al. (Protokoll 2B) [55], according to the manufacturer’s instructions. Briefly, the functional annotation of a gene list, network neighborhood-based entity sets (NESTs) and Gene Ontology Level 2 NESTs were used.

### 4.6. Western Blot Analysis

HL-1 cardiomyocytes were stimulated with C16:1n7, as described above, lysed in RIPA buffers, and ca. 30 μg of protein lysate per line were analyzed using WB, as described previously [8], separated by 10% SDS-PAGE gels and blotted onto a PVDF membrane. Proteins were detected using antibodies directed against extracellular signal-regulated kinases 1/2 (ERK1/2) (Anti-Phospho-p44/42 MAP Kinase (Thr202/204), Anti-p44/42 MAP Kinase (Thr202/204) (Cell Signaling Technology, dilution 1:1000), Forkhead Signalling Antibody Sampler Kit (Cell Signaling Technology, dilution 1:1000) and goat anti-mouse horseradish peroxidase-coupled secondary antibody (Jackson ImmunoResearch, dilution 1:10000)). For detection, chemiluminescent reagents (ECL Western blotting reagents, GE Healthcare, Chicago, USA) were used. Signal densities were analyzed using Image Lab software (Bio-Rad, Hercules/Kalifornien, USA, version 6.0.1).

### 4.7. Gene Expression Analysis

Total RNA from PCMs and HL-1 cells was isolated using the RNeasy Micro Kit according to the manufacturer’s protocol (Qiagen, Germany) Kit. cDNA was synthesized by reverse transcription using reverse transcriptase, RNasin and dNTPs (all Promega, Madison, Wisconsin, USA). For the analysis of gene expression, a real-time quantitative polymerase chain reaction (qRT-PCR) was performed on a CFX96 Real-Time PCR System (BioRad, Hercules/Kalifornien, USA) using SYBR Green technology. Relative gene expression was calculated by the 2-ΔΔCT method with Heat shock protein 90 α family (*Hsp90ab1*) or *β-actin*, as indicated. The list of primers list is available upon individual request.

### 4.8. PPAR-Specific Gene Expression Analysis

Pathway-focused qRT-PCR analysis of cardiac samples (*n* = 3 per group) was performed (RT^2^ Profiler™ Mouse PPAR Array; Qiagen, Hilden, Germany) according to the manufacturer’s protocol. For each gene, the same threshold was used; CT values >35 were considered as no expression. The array contained a profile of PPAR-specific target genes, reverse transcription controls, intern PCR controls, genomic DNA quality control and several housekeeping genes. Analyses were performed using RT2 Profiler PCR Array Data Analysis software (version 3.5, Qiagen, Hilden, Germany). Relative gene expression was calculated by the 2-ΔΔCt method with HSP90ab1 as a housekeeping gene. A heatmap of expression values (2-ΔΔCt) was created using the web-based software ClustVis and R-script.

### 4.9. Histology

Paraffin-embedded cardiac cross-sections were deparaffinized and collagen fibers were stained with Picrosirius red, as described before [8]. Digital images of sections were captured using a slide scanner (Aperio CS2, Leica Biosystems, Nussloch, Germany) and a classifier algorithm (Aperio GENIE, Leica Biosystems, Nussloch) was trained to detect red-stained collagen fibers. Subendocardial and subepicardial collagen content were defined as the proportion of collagen fibers in the whole cardiac tissue.

### 4.10. Statistical Analysis

Data are presented in the figures of this study as mean ± SEM. Statistical analyses were performed using GraphPad Prism 8 (GraphPad Software, San Diego, CA, USA), as described previously [8]. Normally distributed data (according to the Shapiro–Wilk test) were analyzed using a two-tailed unpaired Student’s *t*-test or two-way ANOVA followed by Tuckey post-hoc test, as indicated. A value of *p* < 0.05 was considered statically significant; in the figures, * *p* < 0.05, ** *p* < 0.01, *** *p* < 0.001 and **** *p* < 0.0001. Grubbs’ test was performed to identify outliers.

## Figures and Tables

**Table 1:** Echocardiographic analysis of the mice.

	VEH	C16:1n7	ISO	C16:1n7/ISO
IVSd (mm)	0.6613 ± 0.048	0.6488 ± 0.068	0.6817 ± 0.095	0.6191 ± 0.083
PWd (mm)	0.5818 ± 0.073	0.5626 ± 0.063	0.5639 ± 0.061	0.5567 ± 0.055
LVIDd (mm)	3.717 ± 0.202	3.602 ± 0.207	3.882 ± 0.174	3.890 ± 0.209 *
FS (%)	26.12 ± 5.92	26.67 ± 5.89	24.85 ± 3.87	26.26 ± 2.33
LVM (mg)	74.65 ± 10.9	72.60 ± 5.46	80.92 ± 13.11	81.88 ± 7.51
HW (mg)	113.8 ± 5.95	113.7 ± 10.1	122.6 ± 8.63	121.3 ± 5.72

Final echocardiographic analysis, performed after 22 days of treatment. Values are shown as mean ± standard error of the mean; IVSd, septum thickness during diastole; PWd, posterior wall thickness during diastole; LVIDd, LV internal diameter during diastole; FS, fraction shortening; LVM, left ventricular mass; HW, heart weight. *n* = 8–10 per group; * = *p* < 0.05 C16:1n7 vs. C16:1n7/ISO; 2-way ANOVA (Tukey post-test).

## Data Availability

An RNA-Seq data set is available in the Supplementary Materials.

## Supplementary Materials

The following are available online at https://www.mdpi.com/article/10.3390/ijms222312695/s1.
