# Supplementary material for: Cardioprotective Effects of Palmitoleic Acid (C16:1n7) in a Mouse Model of Catecholamine-Induced Cardiac Damage Are Mediated by PPAR Activation
Source: Int J Mol Sci. 2021 Nov 24;22(23):12695. doi: 10.3390/ijms222312695 (PMC8657733; doi:10.3390/ijms222312695)
Supplement: Supplementary file 1 [file ijms-22-12695-s001.zip › ijms-1434211 supplementary.pdf]

A

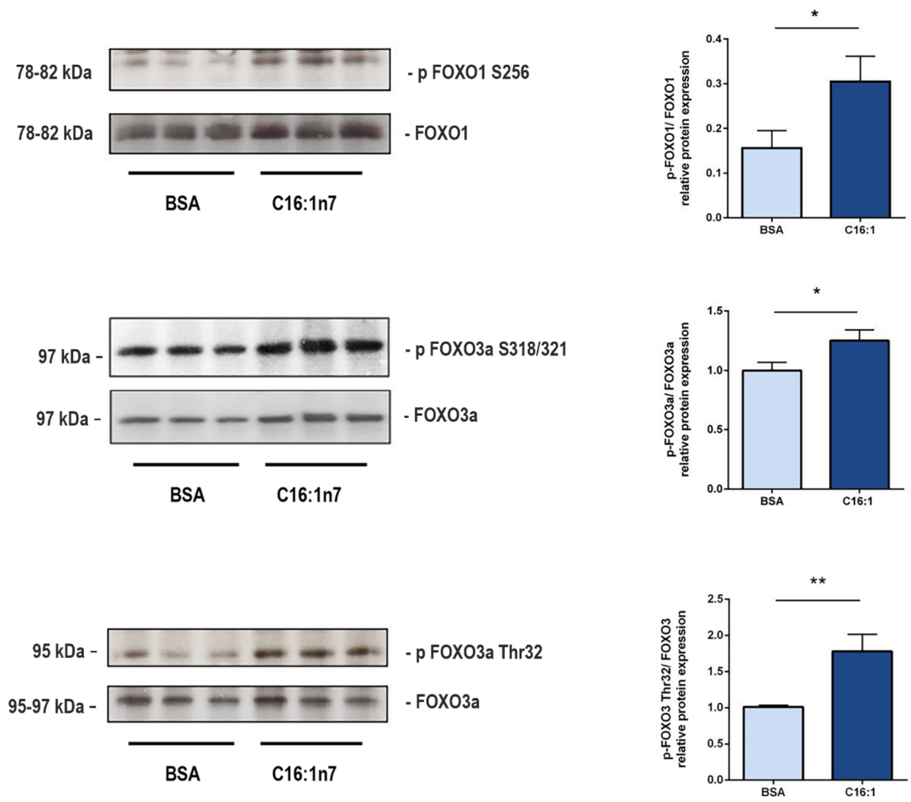

B

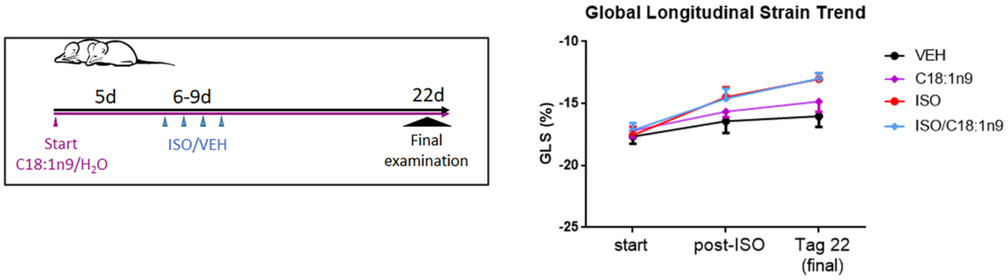

Figure S1. (**A**) Western Blot analysis of FOXO1 and FOXO3 phosphorylation in HL-1 cardiomyocytes, stimulated with 471 µM C16:1n7 for 30 min. Data are presented as mean ± SEM. $N = 3$, $n = 3$, * $p < 0.05$, ** $p < 0.01$, as analyzed using unpaired ttest. (**B**) C18:1n9 application to mice seems to be less protective against ISO-induced cardiac damage. (**B**) Experimental design. The 129sv wt mice were daily orally supplemented with C18:1n9 or Vehicle, as control. After 5 days (pretreatment), the mice received additionally s. c. ISO/Veh application for 4 consecutive days. At the baseline, day after last ISO application and during the final examination (day 22) the animals underwent echocardiographic analysis. GLS-trend is shown, n= 7-12, control groups as in Figure 3. GLS: global longitudinal peak strain, FOXO: Forkhead-Box-Protein O.
